# Fractionated stereotactic radiotherapy of intracranial postoperative cavities after resection of brain metastases – Clinical outcome and prognostic factors

**DOI:** 10.1016/j.ctro.2024.100782

**Published:** 2024-04-21

**Authors:** L. Hahnemann, A. Krämer, C. Fink, C. Jungk, M. Thomas, P. Christopoulos, J.W. Lischalk, J. Meis, J. Hörner-Rieber, T. Eichkorn, M. Deng, K. Lang, A. Paul, E. Meixner, F. Weykamp, J. Debus, L. König

**Affiliations:** a Department of Radiation Oncology, University Hospital of Heidelberg, Im Neuenheimer Feld 400, 69120 Heidelberg, Germany; b Department of Radiation Oncology, University Hospital of Mainz, Langenbeckstraße 1, 55131 Mainz, Germany; c National Center for Radiation Oncology (NCRO), Heidelberg Institute for Radiation Oncology (HIRO), Im Neuenheimer Feld 400, 69120 Heidelberg, Germany; d Department of Neurosurgery, University Hospital of Heidelberg, Im Neuenheimer Feld 400, 69120 Heidelberg, Germany; e Department of Thoracic Oncology, Thoraxklinik and National Center for Tumor Diseases at Heidelberg University Hospital, Heidelberg, Germany; f Translational Lung Research Center Heidelberg (TLRC-H), Member of the German Center for Lung Research (DZL), Germany; g Department of Radiation Oncology, Perlmutter Cancer Center at New York University Langone Health at Long Island, New York, NY, USA; h Institute of Medical Biometry, University of Heidelberg, Im Neuenheimer Feld 130, 69120 Heidelberg, Germany; i Heavy Ion Therapy Center (HIT), Heidelberg University Hospital, Im Neuenheimer Feld 450, 69120 Heidelberg, Germany; j Clinical Cooperation Unit Radiation Oncology (E050), German Cancer Research Center (DKFZ), Im Neuenheimer Feld 280, 69120 Heidelberg, Germany

**Keywords:** Fractionated stereotactic radiotherapy, Brain metastases, Resection cavity, Radiation-induced contrast enhancements, Immunotherapy

## Abstract

**Background and Purpose:**

After surgical resection of brain metastases (BM), radiotherapy (RT) is indicated. Postoperative stereotactic radiosurgery (SRS) reduces the risk of local progression and neurocognitive decline compared to whole brain radiotherapy (WBRT). Aside from the optimal dose and fractionation, little is known about the combination of systemic therapy and postoperative fractionated stereotactic radiotherapy (fSRT), especially regarding tumour control and toxicity.

**Methods:**

In this study, 105 patients receiving postoperative fSRT with 35 Gy in 7 fractions performed with Cyberknife were retrospectively reviewed. Overall survival (OS), local control (LC) and total intracranial brain control (TIBC) were analysed via Kaplan-Meier method. Cox proportional hazards models were used to identify prognostic factors.

**Results:**

Median follow-up was 20.8 months. One-year TIBC was 61.6% and one-year LC was 98.6%. Median OS was 28.7 (95%-CI: 16.9–40.5) months. In total, local progression (median time not reached) occurred in 2.0% and in 20.4% radiation-induced contrast enhancements (RICE) of the cavity (after median of 14.3 months) were diagnosed. Absence of extracranial metastases was identified as an independent prognostic factor for superior OS (p = <0.001) in multivariate analyses, while a higher Karnofsky performance score (KPS) was predictive for longer OS in univariate analysis (p = 0.041). Leptomeningeal disease (LMD) developed in 13% of patients.

**Conclusion:**

FSRT after surgical resection of BM is an effective and safe treatment approach with excellent local control and acceptable toxicity. Further prospective randomized trials are needed to establish standardized therapeutic guidelines.

## Highlights

• FSRT with 35 Gy in 7 fractions for brain metastases resection cavities is effective.
• Postoperative FSRT is a safe treatment approach with acceptable toxicity.
• Diagnosis of RICE is challenging and should be discussed interdisciplinary.
• Long term clinical and radiological follow-up is essential.

## Introduction

Brain metastases (BM) represent the most frequent intracranial tumor in adults and imply a severe complication of uncontrolled systemic disease [1]. About 15 % to 20 % of cancer patients develop BM during their course of disease [2]. Overall survival (OS) of patients with BM is usually low and does not exceed many years in historical cohorts [3], [4], [5].

Given the improvements in systemic therapy regimes, following diagnosis of intracranial disease survival can be prolonged particularly in the setting of intracranially active systemic agents [6], [7], [8]. Furthermore, due to improvements in systemic therapy and better diagnosis through improved imaging techniques [9], [10] the incidence of BM is increasing [11].

Surgery is an effective treatment for selected patients with limited disease, as well as for patients with mass effects caused by large tumors. Furthermore, surgery is preferred in patients where the diagnosis is not yet clear due to the possibility of histopathological analyses [12], [13]. Nevertheless, without adjuvant therapy, intracranial local progression is at least 50 % [14], [15], [16].

Whole brain radiotherapy (WBRT) historically was the standard of care in effort to reduce the risk of local and distant relapse [4], [14]. The development of more advanced imaging, treatment planning, and radiation equipment has allowed the ubiquitous use of stereotactic radiosurgery (SRS). Data has demonstrated that postoperative single-fraction SRS improves local control (LC) significantly in comparison to observation alone (one-year freedom from local recurrence 72 % vs. 43 %) [16] and significantly lowers neurocognitive impairment relative to WBRT [17]. Furthermore, adjuvant WBRT in patients with a limited number (1–3) of resected BM failed to improve OS compared to SRS [4], [17] and showed a negative effect on health-related quality of life (Qol) [17], [18]. Brown et al. reported poorer LC rates after SRS (61 %) compared with WBRT (81 %) [17]. Three major hypotheses for this result have been posited: First, the prescribed dose was dependent on cavity volume. Thus, to avoid toxicity, the dose was reduced for larger targets (12 Gy for ≥ 30.0 ml cavity volume). Therefore, it is likely that the dose for some of the lesions was too low to achieve satisfactory LC rates. Second, the subtotal resection of 8 % was quite high in patients treated with SRS. Finally, the treatment margins of 1–2 mm may not have been sufficient to cover microscopic disease particularly with the aforementioned variations in surgical results and radiation dose [17], [19].

There are a variety of challenges related to postoperative radiotherapy (RT) for BM including the relatively large volume requiring high-dose radiation, particularly relative to intact metastases, as well as challenges in delineating the target cavity and surgical tract correctly. With an increasing treatment volume, the threshold to develop radiation-induced contrast enhancements (RICE) and clinically evident radiation necrosis (RN) [20] and the effectiveness in relation to the LC [21] decline. Fractionated stereotactic radiotherapy (fSRT) is an alternative treatment approach to SRS [22], [23]. On the one hand, with fractionated dose schedules the risk of RICE can be reduced, especially in larger cavities [24], [25]. Moreover, one-year LC rates of up to 88 % were reported since the biologically effective dose (BED) can be escalated for larger volumes with fSRT [26], [27], [28].

To date, several fundamental questions remain unanswered, including the optimal dose fractionation schedule and the best definition of the target volume, as margins ranging from 1 and 10 mm have been reported [24], [29], [30].

Due to the change in standard of care from postoperative WBRT to postoperative SRS, higher rates of leptomeningeal disease (LMD) have been observed [31]. LMD incidence after SRS to the resection cavity was reported with a one-year risk of 7–31 % [16], [17], [32], [33], while LMD rates after fSRT have been reported between 13–19 % [27], [34], [35], [36].

Newly implemented systemic therapy, including immunotherapy (IT) and targeted therapy (TT), have been approved for a variety of tumor entities and have yielded improved cancer survival. This ongoing development led more often to use of systemic agents parallel to RT [37]. However, little is known regarding the nature and the scale of the potential impact of systemic agents on fSRT/SRS toxicity. Combined IT/TT seems to be a treatment factor associated with the development of RICE, but the clinical evidence is still limited, and prospective studies are lacking [38], [39], [40], [41], [42].

In this study, we evaluated the clinical outcomes from a single-center with 105 patients treated with fSRT to the resection cavity following surgical resection for intracranial metastases. Patient and their treatment related factors for OS and total intracranial brain control (TIBC) were identified. Furthermore, we analyzed clinical variables, including systemic therapy, which are discussed to be associated with the development of RICE.

## Methods

### Patient selection

In this study, all patients were treated at a European tertiary cancer centre between September 2016 and May 2022 and were available for analysis. Patient, treatment, and survival data were obtained from the clinical research database, the nation cancer database and from review of the medical records. Only patients who received fSRT to an intracranial resection cavity treated with 35 Gy in 7 fractions were included in this analysis. The study was primary histology agnostic. The analysis was performed retrospectively and longitudinally throughout the follow-up period. This study was approved by the institutional ethics committee (S-494/2021).

### Treatment planning and delivery

Oncologic treatment recommendations were made in interdisciplinary setting. FSRT was performed with a CyberKnife M6 (Accuray Inc., Sunnyvale, California) system. Immobilization was achieved with a customized thermoplastic mask. The planning scans for fSRT treatment included computed tomography (CT) and high-resolution magnetic resonance imaging (MRI). Following standardized imagine protocols, CT scans and T1-weighted MRI with and without contrast agent both with slice thickness of 1 mm were incorporated. Planning scans were co-registered for target lesion and organs at risk delineation, both image modalities were fused. Dose constraints in nontarget/normal tissue followed the recommendation of the UK Consensus on Normal Tissue Dose Constraints for Stereotactic Radiotherapy [43] and the October 2008 issue of Seminars in Radiation Oncology [44]. Following established institutional guidelines, the following target volumes were generated: (1) the gross tumor volume (GTV) represented any gross residual diseases, (2) the surgical resection cavity along with the surgical tract was marked as clinical target volume 1 (CTV1), while (3) CTV2 was created using a 3 mm isotopic margin from CTV1 including the adjacent meninges. Finally, (4) a planning target volume (PTV) was contoured as CTV2 + 1 mm isotopic margin. According to the department’s protocol and analogous to the ESTRON trial [45] 35 Gy was prescribed in 7 fractions to the surrounding 70 % isodose for all resection cavities in this study. The BM treated before, additionally or after fSRT on resection cavity received doses of 20 Gy, 18 Gy, and 30 Gy in 6 fractions prescribed to the 70 % isodose for tumors with maximum diameter of < 20 mm, 20–30 mm and > 30 mm. As part of the postoperative radiation treatment, the patients received anti-edematous dexamethasone prophylaxis on each day of the treatment with 4 mg.

### Systemic treatment

The systemic treatments, including IT, TT, and chemotherapy (CTx), received during oncologic treatment were recorded in a binary way in the present study. IT and TT were subcategorized. Any dose of systemic therapies given within 14 days before or after fSRT, was recorded and defined as simultaneous.

### Follow-up

Patients were seen at regular three-months intervals or as clinically required. Post-fSRT follow-up consisted of clinical-neurological assessment and contrast enhanced brain MRI. In case of distant progression, images were reviewed in an interdisciplinary setting and surgery and/or reirradiation (SRS, fSRT or WBRT) was offered. Salvage WBRT was recommended for patients with multiple (≥10) distant lesions and/or LMD. Neurological death was documented, if the death was most likely attributable to brain metastases and/or leptomeningeal disease and if there was clinical evidence such as seizures. Patients with no clinical evidence of progressive CNS disease were categorized as non-neurological deaths.

### Outcome

Outcome endpoints were OS, TIBC, LC and RICE. OS was defined as the time interval between the start of fSRT and death whereas TIBC and LC was defined as time between the start of fSRT and the diagnosis of intracranial or local progression based on MRI findings or date of last available MRI without progression.

Local or distant progressive metastases were determined based on the BM response and progression criteria proposed by the Response Assessment in Neuro-Oncology (RANO) group [46], [47]. Lin et al. demonstrated with posttreatment imaging, it may be difficult to determine whether contrast enhancement is due to tumor progression or to treatment-associated effects such as RN. Furthermore, the term of RN itself is not consistently used throughout the literature and therefore homogeneous diagnoses can be challenging. In this study, we used the term “RICE” with every clinically evident RICE lesion suspicious of RN, which was reviewed in an interdisciplinary tumor board. Diagnosis was made using a multistep approach as recommended in the guideline for central nervous system radiation necrosis by the Deutsche Gesellschaft für Radioonkologie (DEGRO) [48].

Acute (during treatment until 3 months after fSRT) and chronic (>3 months after fSRT) toxic effects were assessed during follow-up. Toxicity was graded as stated in the Common Terminology Criteria for Adverse Events (CTCAE), version 5.0.

### Statistical analyses

Descriptive statistics were used for baseline analyses. Continuous variables are given as median (with range or Q1Q3) values and categorial variables as absolute or relative frequencies. Follow-up, including OS, TIBC and LC were summarized through the Kaplan-Meier method. Univariate and multivariate cox regression analyses were performed on potential prognostic baseline characteristics for OS and TIBC and carried out with the corresponding hazard ratio (HR), confidence interval (95 %-CI) and p-value. Due to the low number of cases, cox proportional hazard analyses were not fitted for LC nor for RICE. RICE statistics were analyzed with Mann-Whitney-U- and chi-square test. To avoid immortal time bias stemming from the delayed onset of RICE, a Cox proportional hazards model with RICE as a time-dependent covariate was fitted to investigate the association between RICE and OS. This analysis was supplemented by a landmark analysis. P-value ≤ 0.05 was considered to indicate a statistically significance. Statistical analyses were carried out with SPSS (version 28, SPSS, Chicago, IL) and R (version 4.3.2, r-project.org).

## Results

### Patient characteristics

We retrospectively analyzed 105 patients with a total of 145 treatment sessions on 293 lesions including 113 cerebral resection cavities. Median patient age at fSRT was 60 years (range: 32–89 years). A total of 57 female (54.3 %) and 48 male (45.7 %) patients were included. The most frequent primary malignancies in this cohort were lung cancer (40.9 %), breast cancer (21.9 %) and colorectal cancer (9.5 %). A total of 35 (33.3 %) patients had synchronous BM diagnosed with their primary tumor. At time of fSRT to the resection cavity more than half of the patients had uncontrolled extracranial disease (55.2 %), meaning treatment naïve or progressive disease and 53 (50.5 %) had extracranial metastases. The baseline Karnofsky Performance Scale (KPS) was assessed directly before fSRT: The median KPS score was 80 % (Q1-Q3: 70–90 %). The patient characteristics are listed in Table 1.

**Table 1:** Patient Characteristics.

Characteristic		n = 105	%
Patients/Cavities (resected BM)		105/113	
Gender			
	male	48	45.7
	female	57	54.3
Age at fSRT on resection cavity, y			
	median (min–max)	60 (32–89)	
Primary cancer site			
	adeno-NSCLC	30	28.6
	breast cancer	23	21.9
	non-adeno-NSCLC	11	10.5
	colorectal cancer	10	9.5
	renal cell cancer	8	7.6
	melanoma	6	5.7
	SCLC	2	1.9
	others	15	14.3
Initial BM			
	yes	35	33.3
	no	70	66.7
KPS at fSRT			
	median (Q1-Q3)	80 (70–90)	
KPS stratified			
	90–100	35	33.3
	70–80	61	58.1
	≤60	9	8.6
Metastases outside CNS at fSRT			
	yes	53	50.5
	no	52	49.5
Extracranial disease at fSRT			
	controlled	47	44.8
	not controlled	58	55.2
IT/TT simultaneous to fSRT^a^			
	yes	36	34.3
	no	69	65.7
CTx simultaneous to fSRT^b^			
	yes	23	21.9
	no	82	78.1

Abbreviation: BM, brain metastases, fSRT, fractionated stereotactic radiotherapy; y, years; NSCLC, non-small-cell lung cancer; SCLC, small-cell lung cancer; KPS, Karnofsky Performance Status; CNS, central nervous system; IT, immunotherapy; TT, targeted therapy; VEGF, vascular endothelial growth factor; HER2, human epidermal growth factor receptor 2; PI3K, phosphoinositide-3-kinase; CDK, cyclin-dependent kinase; PARP, poly ADP-ribose polymerase; CTx, chemotherapy. ^a^ Any dose of immunotherapies given within 14 days before or after fSRT; ^b^ Any dose of chemotherapies given within 14 days before or after fSRT.

Altogether, 36 (34.3 %) patients received simultaneous IT/TT, 23 (21.9 %) patients received simultaneous CTx (mostly carboplatin; n = 16, 70.0 %) and 18 patients (17.1 %) received both. While the majority of patients received checkpoint inhibitors like pembrolizumab (with a half-life of 22 days), there were also instances of IT/TT with shorter half-lives, such as Axitinib (VEGF-inhibitor), Alectinib (ALK-inhibitor), and Olaparib (PARP-inhibitor). Supplementary Table 1, Table 2 is detailing the distribution of IT/TT and CTx.

### Treatment characteristics

Three patients (2.9 %) had fSRT/SRS on BM elsewhere before fSRT on resection cavity. At the same time as fSRT on resection cavity, 47 patients (44.8 %) had fSRT/SRS in median of one (Q1-Q3: 1–3) additional BM. Median PTV volume of the resection cavities was 22.3 ml (Q1-Q3: 15.0–32.7 ml) and accumulated PTV within all radiated BM and cavities per patient was 24.9 ml (Q1-Q3: 17.4–36.4 ml). Median RT time was 27 min (Q1-Q3: 23–33 min). Detailed treatment characteristics are illustrated in Table 2.

**Table 2:** Treatment and Radiotherapy Characteristics.

Characteristic		n = 105	%
Prior fSRT/SRS on other BM			
	yes	3	2.9
	no	102	97.1
Combined fSRT/SRS on additional BM			
	yes	47	44.8
	no	58	55.2
Time from resection to fSRT, d			
	median (Q1-Q3)	36 (28–48)	
No. of resected BM			
	1	97	92.4
	2	6	5.7
	3	2	1.9
Collimator type			
	fix	4	3.8
	IRIS	24	22.9
	MLC	77	73.3
fSRT time, min			
	median (Q1-Q3)	27 (23–33)	
PTV volume cavity, ml			
	median (Q1-Q3)	22.3 (15.0–32.7)	
PTV volume cum., ml^a^			
	median (Q1-Q3)	24.9 (17.4–36.4)	

Abbreviation: fSRT, fractionated stereotactic radiotherapy; SRC, stereotactic radiosurgery; BM, brain metastasis; d, days; no., numbers; MLC, multi-leaf-collimator; min, minutes; PTV, planning target volume; ml, milliliters, cum., cumulative. ^a^ Cumulative PTV volume of all brain metastases treated with fSRT/SRS per patient.

### Oncological endpoints

Median follow-up from fSRT on resection cavity was 20.8 months (Q1-Q3: 14.0–28.8 months). Seven patients received no follow-up MRI. Therefore, relative frequencies concerning progression and RICE are related to the remaining 98 patients.

**Fig. 1:**
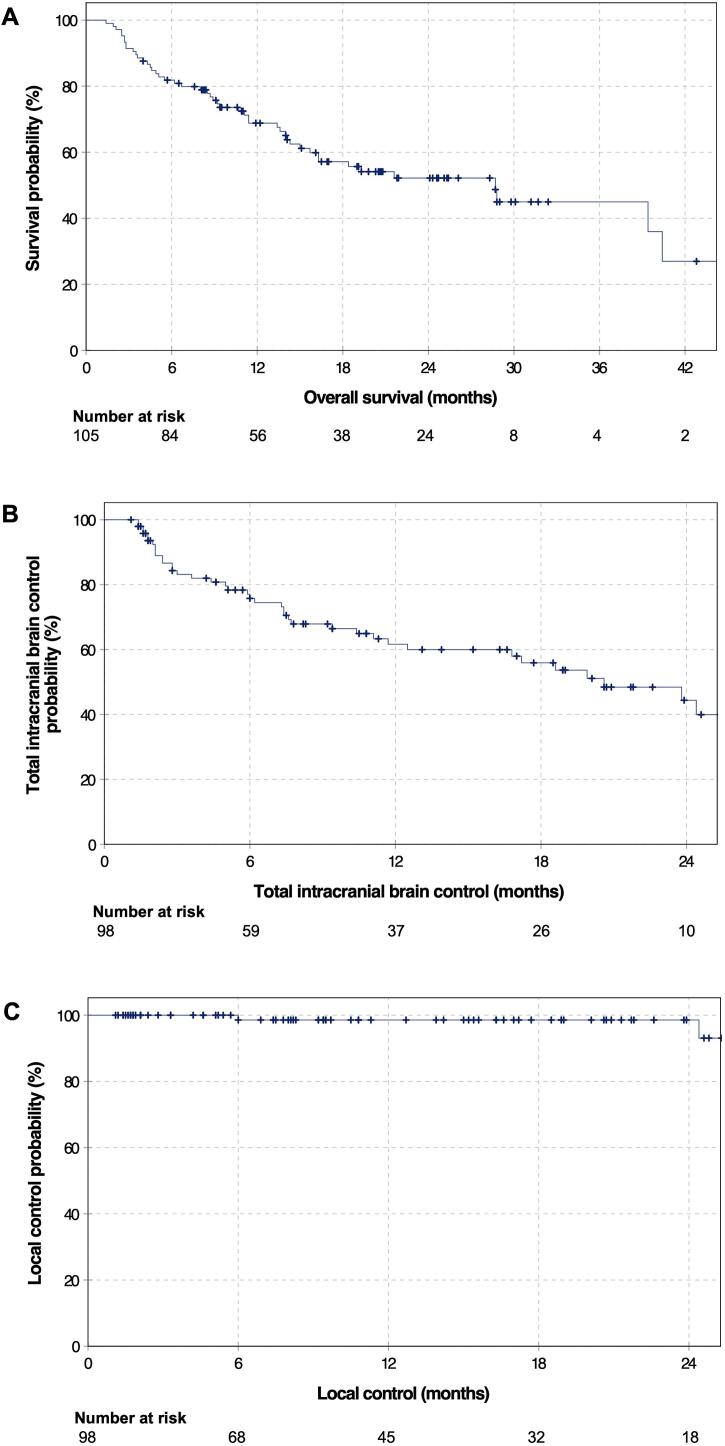
**Kaplan-Meier-Curves**. **A** Overall survival with corresponding numbers at risk. The median overall survival in the cohort (n = 105) was 28.7 months (95 %-CI: 16.9–40.5). **B** Total intracranial brain control with corresponding numbers at risk. The median total intracranial brain control in the cohort (n = 98) was 20.6 months (95 %-CI 13.2–28.0). **C** Local control with corresponding numbers at risk. The median local control in the cohort (n = 98) was not reached yet.

**Supplementary Fig. 1:**
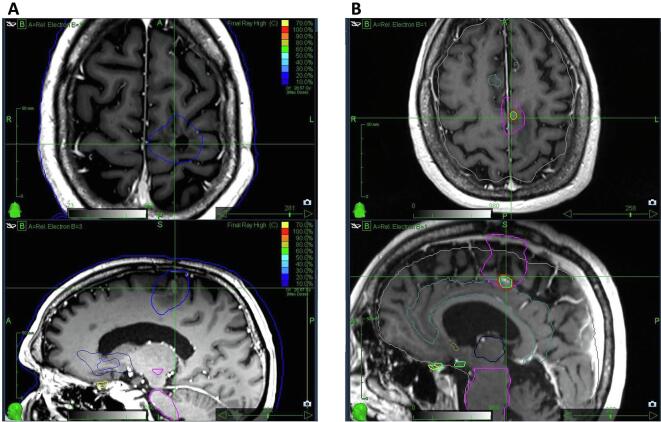
**Local Progression and Radiation-induced Contrast Enhancement.A** Infield local progression in the frontal left hemisphere 6.0 months after fSRT on reception cavity. **B** Infield local progression in the parasagittal left hemisphere 24.4 months after fSRT on resection cavity.

After cavity fSRT, 39 patients (37.1 %) developed intracranial progression, of which 37 (94.9 %) only had a distant progression and two (5.1 %) had both local and distant progression. 11 (28.2 %) of those 39 patients with CNS progression had multifocal progression with ≥ 10 BM. The remaining 28 (71.8 %) patients developed a median of two progressive metastases (Q1-Q3: 1–3). The median TIBC was 20.6 months (95 %-CI 13.2–28.0). The median LC was not reached yet. The LC at six months, one and two years was 98.6 %, 98.6 %, and 93.1 % respectively. In Fig. 1 TIBC and LC are shown. The two patients with local progression had those totally in-field and without dural involvement (Supplementary Fig. 1).

A second course of fSRT/SRS was performed in 24 (61.5 %) patients with progressive metastases (n = 39). During the course of their disease, 16 patients (15.2 %) were treated with salvage WBRT. In Table 3 detailed data on outcome and salvage therapies are provided.

**Table 3:** Outcomes, Radiation-induced contrast enhancement and Leptomeningeal disease.

Characteristic		n = 105/98^a^	%
Outcomes			
CNS progression			
no		59	56.2
yes		39	37.1
	distant	37	94.9
	distant + local	2	5.1
no follow-up image		7	6.7
Number of progressive BM			
	median (Q1-Q3)	2 (1–3)	
	multiple^b,c^	11	11.2
Further fSRT/SRS of BM after fSRT of resection cavity^b^			
	yes	24	24.5
	no	74	75.5
Time from fSRT to next fSRT/SRS, mo			
	median (Q1-Q3)	9.0 (5.5–21.2)	
Salvage-WBRT			
	yes	16	15.2
	no	89	84.8
Time from fSRT to Salvage-WBRT, mo			
	median (Q1-Q3)	3.9 (2.9–8.4)	
Neurological death			
	likely	19	18.1
	no or alive at last contact	61	58.1
	no clinical information	25	23.8
Radiation-induced contrast enhancement			
RICE			
	yes	24	22.8
	no	74	70.5
	no follow-up image	7	6.7
RICE localization^b^			
	resection cavity	16	16.3
	other radiated BM	4	4.1
	both	4	4.1
Time from fSRT to RICE in resection cavity, mo			
	median (Q1-Q3)	14.3 (10.1–20.9)	
Clinical RICE symptoms (CTCAE grading), n = 20			
	Grade 1	2	10.0
	Grade 2	11	55.0
	Grade 3	5	25.0
	Grade 4	2	10.0
	Grade 5	0	0
Therapy for RICE in resection cavity, n = 20			
	no intervention	2	10.0
	Dexa only	7	35.0
	Dexa + Bevacizumab	10	50.0
	Dexa + Bevacicumab + OP	1	5.0
IT/TT simultaneous to fSRT^d^, n = 20			
	yes	7	35.0
	no	13	65.0
CTx simultaneous to fSRT^e^, n = 20			
	yes	3	15.0
	no	17	85.0
Leptomeningeal disease^b^			
	yes	13	13.3
	no	85	86.7
Time from surgery to LMD, mo			
	median (Q1-Q3)	4.6 (3.6–9.6)	
Time from fSRT on cavity to LMD, mo			
	median (Q1-Q3)	3.1 (2.6–8.1)	

Abbreviation: CNS, central nervous system; BM, brain metastasis; fSRT, fractionated stereotactic radiotherapy; SRS, stereotactic radiosurgery; mo, months; WBRT, whole brain radiotherapy; RICE, radiation-induced contrast enhancement; CTCAE, Common Terminology Criteria for Adverse Events; Dexa, Dexamethason; IT, immunotherapy; TT, targeted therapy; CTx, chemotherapy; LMD, leptomeningeal disease. ^a^ 105 patients were included into the survival analysis, 7 patients did not have follow-up MRI and therefore are not included into progression analysis. ^b^ Relative frequencies are related to the 98 patients with follow-up MRI. ^c^ ≥ 10 progressive brain metastases. ^d^ Any dose of immunotherapies given within 14 days before or after fSRT. ^e^ Any dose of chemotherapies given within 14 days before or after fSRT.

During follow-up, 47 (44.8 %) patients had died, 19 (40.4 %) of them likely due to neurological causes. The median OS from cavity fSRT was 28.7 months (95 %-CI 16.9–40.5). OS rates at six months, one and two years were 80.9 %, 68.8 % and 52.2 %. Kaplan-Meier survival curve of OS is presented in Fig. 1.

### Prognostic factors associated with OS and TIBC

OS was not significantly different between patients who did or did not receive IT/TT during course of disease (p = 0.124) or simultaneous to fSRT (p = 0.090). Treatment with CTx during course of disease (p = 0.819) or simultaneous to fSRT (p = 0.448) did not significantly influence OS either.

**Supplementary Fig. 2:**
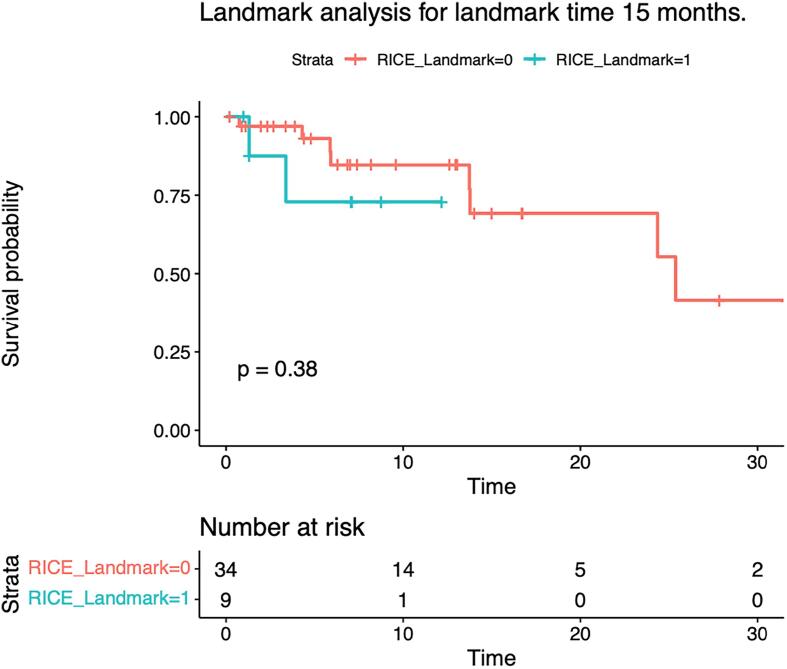
**Landmark Analysis.** Analysis of overall survival in patients with (1) and without (0) RICE with 15-months landmark analysis with corresponding numbers at risk. Survival was estimated using the Kaplan-Meier method in each group. Groups were compared using the log-rank-test (no RICE vs RICE, HR 2.110; 95%-CI 0.386-11.546; p= 0,38. RICE, radiotherapy-induced contrast enhancement.

Univariate analyses identified a higher KPS (HR 0.973; 95 %-CI 0.948–––0.999; p = 0.041), absence of extracranial metastases (HR 0.260; 95 %-CI 0.135–0.501; p= <0.001) and a controlled primary tumor at time of fSRT on cavity (HR 0.504; 95 %-CI 0.272–0.933; p = 0.029) as significant prognostic factors for OS. In multivariate analyses, the absence of extracranial metastases (HR 0.272; 95 %-CI 0.128–––0.576; p= <0.001) was identified as independent prognostic factors for superior OS. RICE was analyzed in a Cox proportional hazards model as a time-dependent covariate for OS but did not show a significant result (HR 1.330; 95 %-CI 0.505–3.503; p = 0.564). The supplementary landmark analysis was consistent with this analysis and as shown by the exemplary landmark time at 15 months, where the group distribution was favorable (Supplementary Figure 2).

For TIBC, the absence of extracranial metastases at time of fSRT (HR 0.485; 95 %-CI 0.256–0.917; p = 0.026) and no further fSRT/SRS for other BM at time of fSRT on cavity (HR 0.425; 95 %-CI 0.222–0.813; p = 0.010) were significantly associated with superior TIBC. Both factors remained significant in multivariate analyses (p = 0.021 and p = 0.019). Furthermore, IT/TT simultaneous to fSRT was identified as significant prognostic factor for TIBC in univariate and multivariate analysis (p = 0.046 and p = 0.024). IT/TT given during course of disease seemed to be associated with improved TIBC, although this difference did not reach statistical significance (HR 0.559; 95 %-CI 0.278–1.124; p = 0.102). The different subcategories of IT/TT could not be analyzed with cox regression due to the small number of cases. All prognostic factors considered for uni- und multivariate cox regression are listed in Table 4, Table 5.

**Table 4:** Analyzed Factors in Univariate and Multivariate Cox Regression with the corresponding HR and P-values for Overall Survival.

Factor analyzed	HR	95 % CI	P-value
Univariate Cox Regression			
Gender (female* vs. male)	0.751	0.421–1.341	0.333
Time from surgery to fSRT on cavity, d	0.992	0.973–1.011	0.416
IT/TT during course of disease (yes* vs. no)	0.611	0.327–1.145	0.124
IT/TT simultaneous to fSRT^a^ (yes* vs. no)	0.566	0.293–1.094	0.090
CTx during course of disease (yes* vs. no)	0.924	0.469–1.819	0.819
CTx simultaneous to fSRT^b^ (yes* vs. no)	0.731	0.326–1.640	0.448
Initial BM (yes* vs. no)	0.837	0.438–1.599	0.590
Age at fSRT on resection cavity, y	1.024	0.998–1.050	0.067
Age stratified (≥60* vs. < 60)	1.659	0.928–2.966	0.088
Extracranial disease at fSRT (controlled* vs. uncontrolled)	0.504	0.272–0.933	0.029
Absent Extracranial Metastases at fSRT (yes* vs. no)	0.260	0.135–0.501	<0.001
KPS at fSRT on resection cavity	0.973	0.948–0.999	0.041
KPS stratified (≥80 %* vs. < 80 %)	0.524	0.266–1.031	*0.061*
Prior fSRT/SRS (yes* vs. no)	1.844	0.445–7.651	0.399
No combined fSRT/SRS on other BM (yes* vs. no)	0.639	0.359–1.136	0.127
RT time	1.011	0.980–1.044	0.482
PTV volume cavity, ml	1.006	0.993–1.020	0.370
PTV volume stratified, ml (≥22.3* vs. < 22.3)	1.164	0.652–2.079	0.608
RICE^c^	1.330	0.505–3.503	0.564

Multivariate Cox Regression			
Extracranial disease at fSRT (controlled* vs. uncontrolled)	0.765	0.709–2.595	0.356
Absent Extracranial Metastases at fSRT (yes* vs. no)	0.272	0.128–0.576	<0.001
KPS at fSRT on resection cavity	0.976	0.949–1.004	0.090

Abbreviation: HR, hazard ratio; CI, confidence interval; fSRT, fractionated stereotactic radiotherapy; IT, immunotherapy; TT, targeted therapy; CTx, chemotherapy; BM, brain metastasis; y, years; CNS, central nervous system; KPS, Karnofsky Performance Status; SRS, stereotactic radiosurgery; RT, radiotherapy; PTV, planning target volume; ml, milliliters. RICE, radiotherapy-induced contrast enhancement ^a^ Any dose of immunotherapies given within 14 days before or after fSRT. ^b^ Any dose of chemotherapies given within 14 days before or after fSRT. ^c^ Time-dependent analysis.

**Table 5:** Analyzed Factors in Univariate and Multivariate Cox Regression with the Corresponding HR and P-values for Total Intracranial Brain Control.

Factor analyzed	HR	95 % CI	P-value
Univariate Cox Regression			
Gender (female* vs. male)	1.459	0.757–2.812	0.259
Time from surgery to fSRT on cavity, d	1.001	0.983–1.018	0.934
IT/TT during course of disease (yes* vs. no)	0.559	0.278–1.124	0.102
IT/TT simultaneous to fSRT^a^ (yes* vs. no)	0.481	0.234–0.988	0.046
CTx during course of disease (yes* vs. no)	1.208	0.532–2.739	0.652
CTx simultaneous to fSRT^b^ (yes* vs. no)	0.471	0.184–1.205	0.116
Initial BM (yes* vs. no)	0.965	0.501–1.859	0.916
Age at fSRT on resection cavity, y	0.987	0.960–1.016	0.381
Age stratified (≥60* vs. < 60)	0.910	0.480–1.724	0.772
Extracranial disease at fSRT (controlled* vs. uncontrolled)	0.844	0.449–1.587	0.599
Absent Extracranial Metastases at fSRT (yes* vs. no)	0.485	0.256–0.917	0.026
KPS at fSRT on resection cavity	0.995	0.965–1.026	0.739
KPS stratified (≥80 %* vs. < 80 %)	0.958	0.475–1934	0.905
Prior fSRT/SRS (yes* vs. no)	0.993	0.135–7.332	0.995
No combined fSRT/SRS on other BM (yes* vs. no)	0.425	0.222–0.813	0.010
RT time	1.024	0.990–1.059	0.164
PTV volume cavity, ml	1.002	0.987–1.017	0.785
PTV volume stratified, ml (≥22.3* vs. < 22.3)	0.740	0.394–1.393	0.351

Multivariate Cox Regression			
Absent Extracranial Metastases at fSRT (yes* vs. no)	0.465	0.243–0.892	0.021
Combined (f)SRT on other BM (yes* vs. no)	0.459	0.239–0.882	0.019
IT/TT simultaneous to fSRT^a^ (yes* vs. no)	0.434	0.210–0.898	0.024

Abbreviation: HR, hazard ratio; CI, confidence interval; fSRT, fractionated stereotactic radiotherapy; IT, immunotherapy; TT, targeted therapy; CTx, chemotherapy; BM, brain metastasis; y, years; CNS, central nervous system; KPS, Karnofsky Performance Status; SRS, stereotactic radiosurgery; RT, radiotherapy; PTV, planning target volume; ml, milliliters; ^a^ Any dose of immunotherapies given within 14 days before or after fSRT. ^b^ Any dose of chemotherapies given within 14 days before or after fSRT. ^c^ Cumulative PTV volume of all brain metastases treated with fSRT/SRS per patient.

### Toxicity

For acute and chronic treatment dependent toxicity 98 patients and 83 patients were evaluable, respectively. Most frequent acute and chronic toxicity was mild grade 1–2 alopecia (39 % and 6 %) and fatigue (39 % and 29 %). Only seizures (3 %) and cerebral edema (1 %) were reported as acute grade 3 toxicity, whereas long term grade 3 side effects were ataxia (1 %), cerebral edema (1 %), seizure (5 %) and decreased vision (1 %). The patient with acute grade 3 cerebral edema suffered from a hemorrhaged resection cavity before fSRT, was treated with dexamethasone due to clinical deterioration (aphasia) and developed LMD 1.8 months after fSRT on the resection cavity. Two of the four patients with grade 3 seizures as chronic toxicity, already suffered from them before first follow-up and are also included in the acute grade 3 toxicities. All other patients who developed chronic grade 3 toxicity were also diagnosed with distant progression after fSRT and received WBRT as salvage therapy. Detailed acute and chronic adverse events data are provided in supplement Table 3.

### Radiation-induced contrast enhancement

RICE developed in 20 patients (20.4 %). The median time between fSRT on resection cavity and diagnosis of RICE on MRI was 14.3 months (Q1-Q3: 10.1–20.9 months). Of those 20 patients with RICE, two patients (10.0 %) were asymptomatic with no intervention needed (CTCAE Grade 1), 11 (55.0 %) developed moderately symptomatic RICE with treatment indication (CTCAE Grade 2), five (25.0 %) had severe symptoms which required additional medical treatment (CTCAE Grade 3) and two (10.0 %) experienced life-threatening symptoms with urgent intervention indicated (CTCAE Grade 4).

In 35.0 % (n = 7), treatment with corticosteroids was sufficient. Ten patients (50.0 %) received corticosteroids and bevacizumab. In addition to corticosteroids and bevacizumab, one patient (5.0 %) underwent surgery, which confirmed the histopathological diagnosis of RN. At time of fSRT 7 (35.0 %) patients had simultaneous IT/TT and 15 (75.0 %) had taken IT/TT at any time during course of disease. Table 3 illustrates further data on RICE. Using the chi-square test there was no significant difference between the frequency of RICE occurrence between patients treated simultaneously with IT/TT (p = 0.940) or CTx (p = 0.407) at fSRT or not. The Mann-Whitney-*U* test revealed that PTV volume was significant larger in patients that developed RICE than those without RICE (median PTV volume 29.5 ml vs. 20.8 ml; p = 0.025). Patient age at time of fSRT (p = 0.119), time between surgery and fSRT in days (p = 0.927) and fSRT duration in minutes (p = 0.445) showed no significant correlation to the development of RICE.

### Leptomeningeal disease

Leptomeningeal disease developed in 13 patients (13.3 %), as presented in Table 3. The median time between fSRT and diagnosis of LMD was 3.1 months (Q1-Q3: 2.6 – 8.1 months)*.* The most frequent primary tumor in this subgroup was HER2 positive breast cancer (n = 7, 53.8 %).

## Discussion

In this retrospective study, the outcome of 105 patients treated with postoperative fSRT to the resection cavity was evaluated. We demonstrate the use of a 35 Gy in 7 fractions treatment regime to be a highly effective approach leading to 98.6 % LC at one year. The rate of RICE was 20.4 % and the rate of LMD was 12.4 %. OS was superior with absence of extracranial metastases, controlled primary tumor and higher KPS score at time of fSRT.

Historically, resection cavities of patients with BM were treated with WBRT. As previous studies have shown, WBRT does not prolong OS [17] and is associated with greater decline in neurocognitive function, such as learning and memory functions [49]. It is formally known that after total (macroscopic) surgical resection microscopic remnant cells remain around the resection cavity. Mahajan et al. demonstrated in a phase 3 study that freedom from local recurrence at one-year was higher after postoperative SRS than after observation only [16]. To date, more and more institutions use local RT but still there is an ongoing discussion regarding the best treatment concept.

In effort to draw conclusions about the efficacy of fSRT as a local treatment, LC rates are of particular interest. In this study only two patients developed local progression. The LC at six months, one and two years was 98.6 %, 98.6 %, and 93.1 % respectively (Fig. 1). These results are favorable in comparison with other retrospective studies in which the LC rates after fSRT were reported between 84 – 88 % [27], [39], [50], [51], [52]. In those studies, the median PTV was between 16.8 and 24.9 ml, which is comparable to a median PTV of 22.3 ml in our analysis. Because of the low case number of local progression in this study, no further statistical analysis of potential prognostic factors was carried out.

The median TIBC was 20.6 months and the one-year TIBC was 61.6 % with 39 patients developing distant intracranial recurrences (Fig. 1). In this study TIBC, including both control over distant and local progression together, was reviewed because of the expected small difference between distant and intracranial failure due to the low impact of local progression. Many studies only report distant control so the following comparison need to be considered with caution. Specht et al. retrospectively reviewed 46 patients treated with 35 Gy in 7 fractions to the resection cavity, of which 57 % showed cranial progression and one-year distant control was 48 % [26] Other studies reached one-year distant control rates between 50.9 and 63 % [52], [53], [54]. It should be noted that these data are from relatively small cohorts and primary tumor therapy has evolved since then, with systemic treatment offering a great impact on distant intracranial control. In this study, IT/TT and CTx (during course of disease or simultaneous to fSRT) was not clearly associated with different OS, although there was a trend towards better OS when IT/TT was administered simultaneous to fSRT (Table 4). For TIBC, simultaneous IT/TT with fSRT was identified as prognostic factor for superior TIBC. This may be in part due to the heterogeneity in the primary tumor histology and applied systemic treatment. Nevertheless, our analysis showed that fSRT as a local treatment offers at least similar local and distant control rates when compared to SRS [17], [49], [55], [56].

The development of distant BM as well as OS is primarily determined by extracranial factors and therapy. In randomized trails, radiotherapeutical intervention, including WBRT and SRS, did not prove consistent evidence of OS improvement compared with other interventions and/or observation [3], [4], [14], [17]. Andrew et al. proved OS improvement for patients treated with WBRT and stereotactic boost vs. WBRT alone (median 6.5 vs. 4.9 months, p = 0.0393) [57]. Patchell et al. demonstrated improved OS for patients after BM resection followed by WBRT compared to WBRT alone (median 40 vs. 15 weeks) [12]. Nevertheless, this is data from three decades ago and given the significant progress in oncological therapies in the recent years, OS is expected to have improved. To address the recent development in systemic therapies, as well as neurosurgical and radiosurgical approaches, Minniti et al. provided a review about the status of cavity irradiation based on studies published between 2012 and 2020. In 16 studies using 12 to 20 Gy in one fraction the one-year OS was 50–70 % and the one-year OS was 62–77 % in fractionated treatment regimens using 25–35 Gy in 5 fractions [56]. Thus, the one-year OS of 68.8 % in our study is comparable to the existing current literature.

With excellent LC rates and comparable OS rates, the effectiveness of fSRT is confirmed in the present study. Of no less importance is the Qol and safety of the applied treatment. Eitz et al. evaluated the effect of fSRT to the resection cavity in a multi-institutional analysis including 558 patients. They found 2.8 % grade 3 toxicity 6 months after fSRT and 4.1 % during the further follow-up time, without specifying what specific toxicities occurred [27]. In our study, acute and chronic grade 3 toxicity including seizures, cerebral edema, ataxia, and decreased vision was reported. Grade 4 toxicities were only caused by RICE and grade 5 toxicity did not occur. Most of the side effects experienced are not pathognomonic for fSRT and may therefore have other causes. Indeed, surgical resection, systemic therapy, cerebral progression, or extracranial factors may also be causative. Therefore, despite utmost caution in data collection, it is possible that the adverse event rate is higher than solely attributed to fSRT alone.

RICE/RN is a common and serious treatment-related side effect following stereotactic RT of BM. Definition, diagnosis, and therapy of RICE/RN is challenging and widely discussed [48]. Physicians are frequently confronted with the evaluation of a contrast enhancing lesion (CEL) detected during regular follow-up imaging. Not only RICE/RN, but also blood–brain barrier disruptions (BBD) due to tumor progression itself can represent as CEL [58]. Recently, comprehensive guidelines regarding the classification and diagnosis of RICE/RN have been published. In conclusion, a multistep approach for the diagnosis is recommended [48]. 19 patients (19.4 %) in this study developed RICE within the irradiated area. Compared with mostly asymptomatic BBD, which usually develop in the first six months after RT [48], the available literature describes the occurrence of RICE/RN within six to 18 months after RT [38], [59]. In this study, the median time to RICE onset was 14.3 months, which resembles these criteria. Taking the range into account, the shortest latent period from fSRT to RICE was 2.1 months. So called early RICE/RN typically develop in between six months after RT [60], [61]. Based on the dose/volume tolerance guidelines from Emami et al. and the QUANTEC review, 60 Gy EQD2 results in a 5 % rate in five years for brain necrosis [62], [63], [64]. Using the linear quadratic model 35 Gy in 7 fractions and α/β = 2 Gy, EQD2 is 61.25 Gy. The rate of RICE in this study seamed surprisingly high (n = 19, 19.4 %). An explanation is the steep dose gradient of 3–4 Gy/mm with CyberKnife technique and the margins we used, which leads to doses > 60 Gy EQD2 in irradiated surrounding brain tissue. Furthermore, more than half of the patients with RICE were asymptomatic (n = 2, 10.0 %) or had moderate symptoms (n = 11, 55.0 %). RICE seems not to be associated with OS when tested as a time-dependent covariate (p = 0.564) in a Cox proportional hazards model. This is consistent with the results of a supplementary landmark analysis.

The comparison with the literature is difficult because reported RN rates are highly dependent on awareness of the diagnostic criteria. Incidences between 7 % and 24 % are reported after SRS in general [58]. For postoperative irradiated cavities with single-fraction SRS or fSRT, Minniti et al. found 12 months estimated risk rates between 1.5 % and 28 %, respectively, with no observable clinical or treatment differences in the two groups [56]. Since no major differences were observed and fractionated treatment regimens are more commonly used for larger treatment volumes, fSRT seems to be a favorable treatment modality, particularly for larger target volumes [22], [23], [56]. However, additional evidence is needed including prospectively evaluation. An ongoing phase III trial study in the US is currently comparing SRS with fSRT in resected BM in a randomized setting (ClinicalTrials.gov, NCT04114981). It is also important to analyze factors that increase the risk of RICE/RN. Larger target volumes and higher radiation doses have been already addressed as risk factors for the development of RN [20], [38]. Simultaneous administration of systemic therapies (IT/TT) may also influence RN rates [38], [39]. Kowalski et al. examined the risk of symptomatic RN in patients with intact BM treated with SRS and found, that simultaneous IT did not increase the risk of RN (SRS alone vs. SRS + immune checkpoint inhibitors, 7 % vs. 4 %; p = 0.25) [65]. In the presented study, 20 patients developed RICE, of which 7 (35.0 %) received IT/TT concurrent to fSRT. In comparison, 29 (33.7 %) of the patients not diagnosed with RICE (n = 86) received IT/TT concurrently with fSRT. When using the chi-square test, no significant association between IT/TT treatment simultaneous to fSRT and RICE was identified.

In a recent review, the authors concluded that SRS with concurrent checkpoint inhibitor treatment is likely not associated with differences in RN rates [66]. Ideally, however not possible in the rapidly evolving treatment landscape of BM, each existing or new systemic agent should be evaluated to find the best timing and combination of RT and systemic therapy, regarding the risk of adverse event and impact on disease outcomes such as LC [67].

Another complication that was observed in this study was LMD. There is a higher risk of LMD development in patients with multiple BM [33], with infratentorial locations [68], after, piecemeal’’ resection (compared with en bloc resection) [69] and in patients with breast cancer [33], [68], [70]. In this cohort, more than half of the patients who developed LMD were female with HER2 positive breast cancer. In addition, all but one patient had more than one BM at the time of fSRT or developed a distant intracranial progression during follow-up (n = 12, 92.3 %). With those conditions in mind, a LMD rate of 13.3 % still appears to be tolerable. The two landmark studies by Brown and Mahajan, reported a LMD risk of 7.2 % by adding a margin of 2 mm and radiation to the highest isodose [17] and 28.0 % with a 1 mm margin and radiation to the 50 % isodose line [16]. Mahajan et al. did include a meningeal margin for cavities close to the dura but did not incorporate the surgical tract [16]. In our study, meningeal extension and surgical tract was included in the CTV and a total of 4 mm isotopic safety margin was added. In this way, potential microscopic tumor seeding was included within target volumes. However, without established standards, postoperative target volume definition remains complex and varies significantly. A margin of 2–3 mm seems to be associated with superior LC, as reported in several studies [71], [72], [73], [74]. Some studies refrain from using any margins [69] or did not find an association or margins with better LC [75], [76]. In a recent consensus guideline, it is recommended to include the surgical tract and in case if the BM abuts the bone flap, the venous sinus, and the pia mater should be included as well [77].

Limitations of the present study naturally include its retrospective nature and mono-institutional patient population. In addition, there was significant heterogeneity in the primary tumor histology and systemic therapy. Dose modifications of systemic therapies were not reported in this study, as only descriptive analysis would be feasible due to the small number of cases. Anyhow, this could be beneficial for further evaluating the association between systemic treatment and RT.

On the other hand, strength of this study is its relatively large cohort size and the homogenous treatment regime of 35 Gy in 7 fractions prescribed to the 70 % isodose performed with a CyberKnife as well as the relatively long median follow-up period of 21 months in median for this patient cohort. The proportion of patients receiving systemic therapy (IT/TT and CTx) simultaneous to fSRT or during the course of disease corresponds to the proportion of patients receiving those therapies in the general oncologic population. To the best of our knowledge this study is currently the largest single-centre cohort of patients with fSRT for BM tumor beds with 35 Gy in 7 fractions.

## Conclusion

This analysis confirms cavity fSRT with 35 Gy in 7 fractions in patients with BM as a safe and effective treatment method with high LC rates and a relatively low rate of adverse events. Clinical-neurological assessment and imaging (MRI) follow-up is mandatory to identify local and distant progression, as well as adverse events like RICE and offer suitable (salvage-) therapies. Nonetheless, further research needs to be focused on risk-adapted dose and fractionation regime as well as timing of systemic treatment to further improve OS, rates of RICE and Qol including postinterventional cognitive function in future.

## CRediT authorship contribution statement

**L. Hahnemann:** Conceptualization, Formal analysis, Writing – original draft, Visualization. **A. Krämer:** Conceptualization, Writing – review & editing, Supervision. **C. Fink:** Writing – review & editing. **C. Jungk:** Resources. **M. Thomas:** Resources. **P. Christopoulos:** Resources. **J.W. Lischalk:** Writing – review & editing. **J. Meis:** Writing – review & editing. **J. Hörner-Rieber:** Writing – review & editing. **T. Eichkorn:** Writing – review & editing. **M. Deng:** Writing – review & editing. **K. Lang:** . **A. Paul:** . **E. Meixner:** . **F. Weykamp:** . **J. Debus:** Resources, Supervision. **L. König:** Conceptualization, Writing – original draft, Supervision.

## Declaration of Competing Interest

The authors declare the following financial interests/personal relationships which may be considered as potential competing interests: JHR received speaker fees from Pfizer Inc. and ViewRay Inc., travel reimbursement from ViewRay Inc., IntraOP Medical and Elekta Instrument AB as well as grants from IntraOP Medical and Varian Medical Systems outside the submitted work. J.D. received grants from View Ray Inc. J.D. received grants from CRI—The Clinical Research Institute GmbH, Accuray Incorporated, Accuray International Sàrl, RaySearch Laboratories AB, Vision RT limited, Astellas Pharma GmbH, Astra Zeneca GmbH, Solution Akademie GmbH, Ergomed PLC Surrey Research Park, Merck Serono GmbH, Siemens Healthcare GmbH, Quintiles GmbH, Pharmaceutical Research Associates GmbH, Boehringer Ingelheim Pharma GmbH Co, PTW-Freiburg Pychlau GmbH, Nanobiotix A.A. and IntraOP Medical outside the submitted work. LK received speaker fees from Novocure outside the submitted work. The other authors declare that the research was conducted in the absence of any commercial or financial relationships that could be construed as a potential conflict of interest.
