# Supplementary material for: Fractionated stereotactic radiotherapy of intracranial postoperative cavities after resection of brain metastases – Clinical outcome and prognostic factors
Source: Clin Transl Radiat Oncol. 2024 Apr 21;46:100782. doi: 10.1016/j.ctro.2024.100782 (PMC11061678; doi:10.1016/j.ctro.2024.100782)
Supplement: Supplementary Data 1 [file mmc1.docx]

**Fractionated stereotactic radiotherapy of intracranial postoperative cavities after resection of brain metastases – Clinical outcome and prognostic factors.**

Supplementary Table 1 Simultaneous Immunotherapy and Targeted Therapy

| **IT/TT simultaneous to fSRT^a^** | | **t½^b^** | **n= 36/45^c^** |
| --- | --- | --- | --- |
| **Checkpoint inhibitors (31/45)** | Nivolumab | 25d | 4 |
|  | Ipilimumab | 15d | 4 |
|  | Pembrolizumab | 22d | 17 |
|  | Atezolizumab | 27d | 3 |
|  | Durvalumab | 18d | 1 |
|  | Avelumab | 6d | 2 |
| **HER2 inhibitors (6/45)** | Trastruzumab | 28d | 2 |
|  | Pertuzumab | 18d | 2 |
|  | Lapatinib | 24h | 1 |
| **multi-targeted tyrosine kinase inhibitors (2/45)** | Osimertinib | 3h | 1 |
|  | Cabozantinib | 55h | 1 |
| **VEGF inhibitors (3/45)** | Bevacizumab | 19d | 1 |
|  | Axitinib | 4h | 2 |
| **CDK inhibitors (1/45)** | Palbociclib | 29h | 1 |
| **PARP inhibitors (1/45)** | Olaparib | 12h | 1 |
| **Alk tyrosine kinase inhibitors (2/45)** | Brigatinib | 24h | 1 |
|  | Alectinib | 32h | 1 |

Abbreviation: IT, immunotherapy; TT, targeted therapy; fSRT, fractionated stereotactic radiotherapy; t½; half-life; HER2, human epidermal growth factor receptor 2; VEGF, vascular endothelial growth factor; CDK, cyclin-dependent kinase; PARP, poly ADP-ribose polymerase; ALK, anaplastic lymphoma kinase. ^a^ Any dose of immunotherapies given within 14 days before or after fSRT. ^b^ Information about half-life was extracted from the clinic intern drug information service (AID). ^c^ Of the 36 patients that received simultaneous IT/TT with fSRT nine patients had treatment with two different drugs at the same time.
