# Supplementary material for: Fractionated stereotactic radiotherapy of intracranial postoperative cavities after resection of brain metastases – Clinical outcome and prognostic factors
Source: Clin Transl Radiat Oncol. 2024 Apr 21;46:100782. doi: 10.1016/j.ctro.2024.100782 (PMC11061678; doi:10.1016/j.ctro.2024.100782)
Supplement: Supplementary Data 2 [file mmc2.docx]

**Fractionated stereotactic radiotherapy of intracranial postoperative cavities after resection of brain metastases – Clinical outcome and prognostic factors.**

**Supplementary Table 2 Simultaneous Chemotherapy**

| **CTx, simultaneous to fSRT^a^** | | n= 23/28^b^ |
| --- | --- | --- |
| **Platinum analogues (17/28)** | Carboplatin | 16 |
|  | Cisplatin | 1 |
| **Taxan (4/28)** | Paclitaxel | 4 |
| **Pyrimidine antagonists (3/28)** | Capecitabine | 2 |
|  | Gemcitabine | 1 |
| **Vinca Alkaloid (2/28)** | Vinorelbine | 2 |
| **Antracycline (1/28)** | Epirubicin | 1 |
| **Others (1/28)** | FOLFIRI | 1 |

Abbreviation: CTx, chemotherapy; fSRT, fractionated stereotactic radiotherapy. ^a^ Any dose of chemotherapies given within 14 days before or after fSRT. ^b^ Of the 23 patients that received simultaneous CTx with fSRT five patients had treatment with two different drugs at the same time.
