# Supplementary material for: Fractionated stereotactic radiotherapy of intracranial postoperative cavities after resection of brain metastases – Clinical outcome and prognostic factors
Source: Clin Transl Radiat Oncol. 2024 Apr 21;46:100782. doi: 10.1016/j.ctro.2024.100782 (PMC11061678; doi:10.1016/j.ctro.2024.100782)
Supplement: Supplementary Data 3 [file mmc3.docx]

**Fractionated stereotactic radiotherapy of intracranial postoperative cavities after resection of brain metastases – Clinical outcome and prognostic factors.**

**Supplementary Table 3 Acute and Chronic Adverse Events**

|  | Acute^a^ (n= 98) | | | | | Chronic^b^ (n= 83) | | | | |
| --- | --- | --- | --- | --- | --- | --- | --- | --- | --- | --- |
|  | *Grade 1* | *Grade 2* | *Grade 3* | *Grade 4* | *Grade 5* | *Grade 1* | *Grade 2* | *Grade 3* | *Grade 4* | *Grade 5* |
| Alopecia | 35 (36%) | 3 (3%) | - | - | - | 5 (6%) | 0 | - | - | - |
| Amnesia | 1 (1%) | 0 | 0 | - | - | 2 (2%) | 0 | 0 | - | - |
| Ataxia | 6 (6%) | 3 (3%) | 0 | - | - | 6 (7%) | 1 (1%) | 1 (1%) | - | - |
| Cognitive disturbance | 6 (6%) | 0 | 0 | - | - | 5 (6%) | 2 (2%) | 0 | - | - |
| Concentration impairment | 3 (3%) | 0 | 0 | - | - | 6 (7%) | 2 (2%) | 0 | - | - |
| Dizziness | 12 (12%) | 0 | 0 | - | - | 6 (7%) | 0 | 0 | - | - |
| Dysarthria | 1 (1%) | 0 | 0 | - | - | 0 | 2 (2%) | 0 | - | - |
| Dysesthesia | 6 (6%) | 0 | 0 | - | - | 0 | 0 | 0 | - | - |
| Edema cerebral | - | - | 1 (1%) | 0 | 0 | - | - | 1 (1%) | 0 | 0 |
| Fatigue | 32 (33%) | 6 (6%) | 0 | - | - | 19 (23%) | 5 (6%) | 0 | - | - |
| Headache | 8 (8%) | 3 (3%) | 0 | - | - | 10 (12%) | 3 (4%) | 0 | - | - |
| Hemiplegia | 1 (1%) | 3 (3%) | 0 | - | - | 1 (1%) | 3 (4%) | 0 | - | - |
| Nausea | 3 (3%) | 0 | 0 | - | - | 3 (4%) | 2 (2%) | 0 | - | - |
| Paresthesia | 2 (2%) | 1 (1%) | 0 | - | - | 4 (5%) | 1 (1%) | 0 | - | - |
| Seizure | 0 | 0 | 3 (3%) | 0 | 0 | 0 | 0 | 4 (5%) | 0 | 0 |
| Tremor | 1 (1%) | 0 | 0 | - | - | 1 (1%) | 0 | 0 | - | - |
| Vision decreased | - | 10 (10%) | 0 | 0 | - | - | 6 (7%) | 1 (1%) | 0 | - |
| Vomiting | 2 (2%) | 0 | 0 | 0 | 0 | 0 | 0 | 0 | 0 | 0 |
| Asymptomatic | 22 (22%) | | | | | 22 (27%) | | | | |
| Symptoms unkown | 2 (2%) | | | | | 7 (8%) | | | | |

Annotations: Toxicity was graded as stated in the Common Terminology Criteria for Adverse Events, version 5.0^.^ ^a^ acute= during treatment until 3 months after fSRT, n= 98^.^ ^b^ chronic= >3 months after fSRT, n= 83
